# How public health agencies communicate with the public on TikTok under the normalization of COVID-19: A case of 2022 Shanghai's outbreak

**DOI:** 10.3389/fpubh.2022.1039405

**Published:** 2022-11-24

**Authors:** ShaoPeng Che, Shunan Zhang, Jang Hyun Kim

**Affiliations:** ^1^Department of Human-Artificial Intelligence Interaction, Sungkyunkwan University, Seoul, South Korea; ^2^Department of Interaction Science, Sungkyunkwan University, Seoul, South Korea

**Keywords:** COVID-19, public health emergency, public health agency, TikTok, social media, communication strategy, crisis and emergency risk communication, negative binomial regression

## Abstract

**Objective:**

As life with COVID-19 became a norm in 2022, the public's demand for and perception of COVID-19-related information has changed. This study analyzed the performance and responses of Healthy China and the public at various stages of COVID-19 normalization using the crisis and emergency risk communication (CERC) theory.

**Methods:**

This study was based on the 2022 Shanghai COVID-19 outbreak and data from “Healthy China,” the official TikTok account of the National Health Commission of the People's Republic of China (NHCC). First, we divided the Shanghai lockdown into five stages in accordance with the CERC. Second, the videos released by Healthy China were open-coded. Third, to understand the distribution of strategies across the stages, we used counts and percentages to summarize the categorical variables. Fourth, we investigated the distribution of public participation indicators using descriptive statistical analysis. Finally, the relationship between stage and communication strategy was examined using the chi-square test and negative binomial regression.

**Results:**

(1) Healthy China adopted a more flexible approach to communication strategies; (2) new cases per day was the commonly used substrategy for uncertainty reduction; (3) there was a significant difference in the strategies used by Healthy China at different stages; (4) public participation was highest in the pre-crisis period; and (5) the stage had a significant positive impact on the number of views, favorites, likes, and shares.

**Conclusions:**

This research provides insight into effective communication strategies for the government or public health agencies to employ during COVID-19 normalization.

## Introduction

The normalization of COVID-19 means that although most countries and regions worldwide have lifted COVID-19 restrictions having achieved an “end of the epidemic” at the agenda level, from the public health perspective, the epidemic is not over, and the reality is that a large number of people are infected every day and some countries or regions are still implementing different isolation measures. In such a seemingly over, but not over, COVID-19 state, people are eager to return to the normal state of life, travel, and learning that they had before the outbreak in 2020 (1, 2).

In China, the communication strategy of new cases per day starting in 2022 seems synonymous to the “weather forecast.” People may need to speculate on whether the end of the outbreak will affect their future travel plans based on the daily new cases. The public's right to travel is heavily influenced by the local governments' classification of cities as low risk, medium risk, or high risk based on the number of new cases per day in the area. When one's own city is classified as a medium or high risk area due to an increase in the number of new cases per day, leading to an increase in the risk level and control conditions, the public can easily feel pessimistic and distressed. Therefore, it becomes increasingly important to understand if and how the government or public health agency (PHA) is using social media to communicate with the public in the context of the normalization of COVID-19 (3, 4).

As of August 2022, existing studies have focused on traditional text-based social media, such as Twitter. However, few studies have focused on other emerging video-based social media platforms, such as TikTok (5). UNESCO shows that 750 million adults worldwide are illiterate. Undoubtedly, text-based social media is not convenient for illiterate people. However, the emergence of video-based social media has solved this problem to some extent. Although such adults may not be literate, they are able to access information by watching videos and listening to sounds (6). Therefore, when illiterate or semi-literate people use the Internet to acquire knowledge, video media are given higher priority than text media. Furthermore, the most important aspect of risk and crisis communication is to reach a wide audience. In 2021, during the COVID-19 pandemic, TikTok became the world's most visited Internet domain, surpassing Google. In September 2021, TikTok's monthly active users had reached 1 billion. Therefore, TikTok has become an indispensable information source and communication channel between governments, organizations, and the public (5). Hence, this study posed the following research question:

RQ1: How is Healthy China communicating with the public *via* TikTok in the context of the normalization of COVID-19?

Although previous studies have examined data from 2019 to 2020, COVID-19 was still in its early stages, and no complete set of response plans or guidelines have been proposed by the government or PHA. Furthermore, as countries open up their borders and global tourism begins to recover, public fear, anxiety, and perception about COVID-19 and the demand for related information may be different. Therefore, it is necessary to re-measure the indicators related to the government, PHA, and public engagement under the normalization of COVID-19 based on the latest data from social media in 2022.

RQ2: How is the public engaged in Healthy China in the context of COVID-19 normality?

From 2019 to 2020, knowledge of COVID-19 was still in its infancy, with no proven, standardized response guidelines in place. Researchers were unable to determine when the epidemic would end, much less which stage it was currently in, leaving a virtual void in the stage-based analysis. The government, PHA, and public may behave differently at each stage of COVID-19. For example, the public may be very concerned about self-protection-related information early in the outbreak, but this concern may decline during the middle and later stages of the outbreak. In 2022, as Naveen and Gurtoo indicated, there is a greater need for people to be informed about the latest prevention policies through the news, so that they can adjust their planning in time to reduce unnecessary hassles and inconveniences (7). However, these changes may be insignificant when researchers measure the COVID-19 components without phasing. Therefore, proper phasing of COVID-19 and analysis of public engagement are essential. Based on crisis and emergency risk communication (CERC), the following research questions were posed:

RQ3a: How does stage influence communication strategies in the context of COVID-19 normalization?

RQ3b: How does stage influence public engagement in the context of COVID-19 normalization?

RQ3c: How does substrategy influence public engagement in the context of COVID-19 normalization?

Considering the significance of TikTok for illiterate or semi-literate individuals, changes in public perceptions of the outbreak, and the necessity to classify the outbreak into stages, this study explored how Healthy China and the public communicate on TikTok in the context of COVID-19 normalization based on CERC.

### Literature review

#### TikTok users' behavior

With lockdown and social distancing restrictions in place, TikTok has become the primary public information source during the public health emergency (PHE; 1). Risk, crisis, and health information related to PHE are the main components of information obtained by the public (3, 8). Therefore, understanding how the public uses TikTok to create, disseminate, and receive information involves understanding how the government or PHA develops and disseminates communication strategies related to COVID-19.

As of September 2022, scholars found that the types of videos posted by TikTok users were diverse encompassing health information needs, mental state sharing, and vaccine concerns (4). For example, Ostrovsky and Chen analyzed 100 most popular videos related to COVID-19 on TikTok and found that videos made by healthcare professionals received the most likes and shares (9). Basch et al. analyzed top 100 videos on TikTok with the hashtag #Coronavirus and all videos posted by the World Health Organization (10). They found that anxiety and quarantine were the dominant topics within public sentiment and focus, yet < 10% of the videos mentioned how the virus was transmitted, its symptoms, and preventive measures. Baumel et al. examined the top 150 TikTok videos tagged with masks and found that increasing the number of professionals providing medically accurate information on TikTok could improve public health literacy (11). Southwick et al. conducted an encoding analysis of 750 videos tagged with #Coronavirus and found that health-related videos constituted the highest share (12). Finally, Baumel et al. analyzed the top 100 videos with the hashtags #Pfizer and #Moderna, and found that increasing public knowledge and vaccine confidence can boost vaccination rates (13).

The above studies indicate a high public demand for PHE-related information on TikTok, so there is an urgent need for governments or PHAs to develop social media communication strategies based on TikTok users' behavior.

#### Actions of government or PHAs

With increasing public demand for TikTok videos related to COVID-19 (14), it is essential to understand what strategies governments or PHA use to communicate with the public and how the public reacts when faced with different strategies. However, as of August 2022, few studies have explored how governments and PHA have used TikTok to communicate with the public during COVID-19.

Zhu et al. were the first to investigate how 31 provincial health councils in China used TikTok to communicate with the public (15). Focusing on how the content and format of the videos influenced the number of likes, comments, and shares of users, they showed that the public preferred to watch cartoons, documentaries, videos promoting occupational health, and videos providing health knowledge, in addition to videos with original music, mandarin, subtitles, and lasting <60 s. In addition, Chen et al. explored the factors that influenced public engagement in the TikTok account of the National Health Commission of the People's Republic of China (NHCC) between January and April 2020 (5), and indicated that video length, title, dialogic loop, and content type significantly influenced public engagement. Subsequently, with the help of a sentiment lexicon, this study also explored the sentiment-mediating role of video titles, and the results showed that sentiment tendencies could moderate the effect of video length and content type on public engagement. Li et al. investigated how the format, type, and content of videos with COVID-19-related tags posted on TikTok by eight international organizations from November 2019 to May 2022 influenced the number of views, likes, comments, and shares by users (16), and found that dance videos, as well as videos that mentioned risk information and precautionary measures, had higher engagement levels.

In general, it was found that the researchers were all from China and that the video attributes used by the researchers generally referred to the format, type, and content of the video, with the quantitative indicators of public engagement generally consisting of the number of views, likes, comments, and shares. While these studies have done their best to analyze the factors that influence public engagement, they have one common feature; they all use TikTok data from the early stages of the COVID-19 outbreak to explore how governments and PHA use the platform to communicate with the public. In 2019–2020, when the world was still in the awareness stage of COVID-19, governments and PHA focused on providing information, such as telling people what COVID-19 is and how people should respond to the outbreak (17).

In the context of constant variability (18), ups and downs, and the normalization of COVID-19, people seek to return to normal work, school, and life conditions before the epidemic is officially over (19), placing greater pressure on governments and PHA to communicate. Furthermore, the public's perception of COVID-19 and their need for different types of information have also changed dramatically. It is, therefore, necessary to explore how the government or PHA should communicate with the public based on the latest TikTok data and to discuss whether the level of public engagement in 2019–2020 was different from that in 2022 under COVID-19 normalization.

#### Crisis and emergency risk communication

CERC is a five-stage model developed by the Centers for Disease Control and Prevention that integrates risk and crisis communication. CERC solves the conceptual confusion between risk communication and crisis communication by perfectly merging the two into one theoretical framework, and divides the entire emergency cycle into five stages: pre-crisis, initial event, maintenance, resolution, and evaluation. On this basis, it proposes a model that uses different communication strategies at different stages (20).

The first stage is the pre-crisis period, which focuses on the use of risk messages, warnings, and preparedness strategies to inform the public and aid in the monitoring and identification of emerging risks, as well as helping to improve the public's understanding of risk.

The second stage includes strategies for reducing uncertainty, increasing self-efficacy, and providing reassurance. This stage aims to help the public build a broad understanding of the crisis based on the available information by communicating quickly with the public and affected groups.

The third stage is maintenance, which consists of ongoing uncertainty reduction, self-efficacy, and reassurance. This stage is intended to provide the public with a better understanding of the risks involved, as well as to seek support and cooperation in returning to normal life and work.

The fourth stage of the process is resolution, which includes updates on resolution, discussions about causes, and new risks/new understandings of risk. The purpose of this stage is to facilitate a broad discussion of the causes, responsibilities, and adequacy of stakeholders.

Evaluation is the final stage. Its strategy consists of discussions about the appropriateness of response, agreement on causes and new risks, and a new understanding of risks. This stage aims to improve the ability to communicate and respond to future crises by sharing the lessons learned.

## Materials and methods

**Figure 1:**
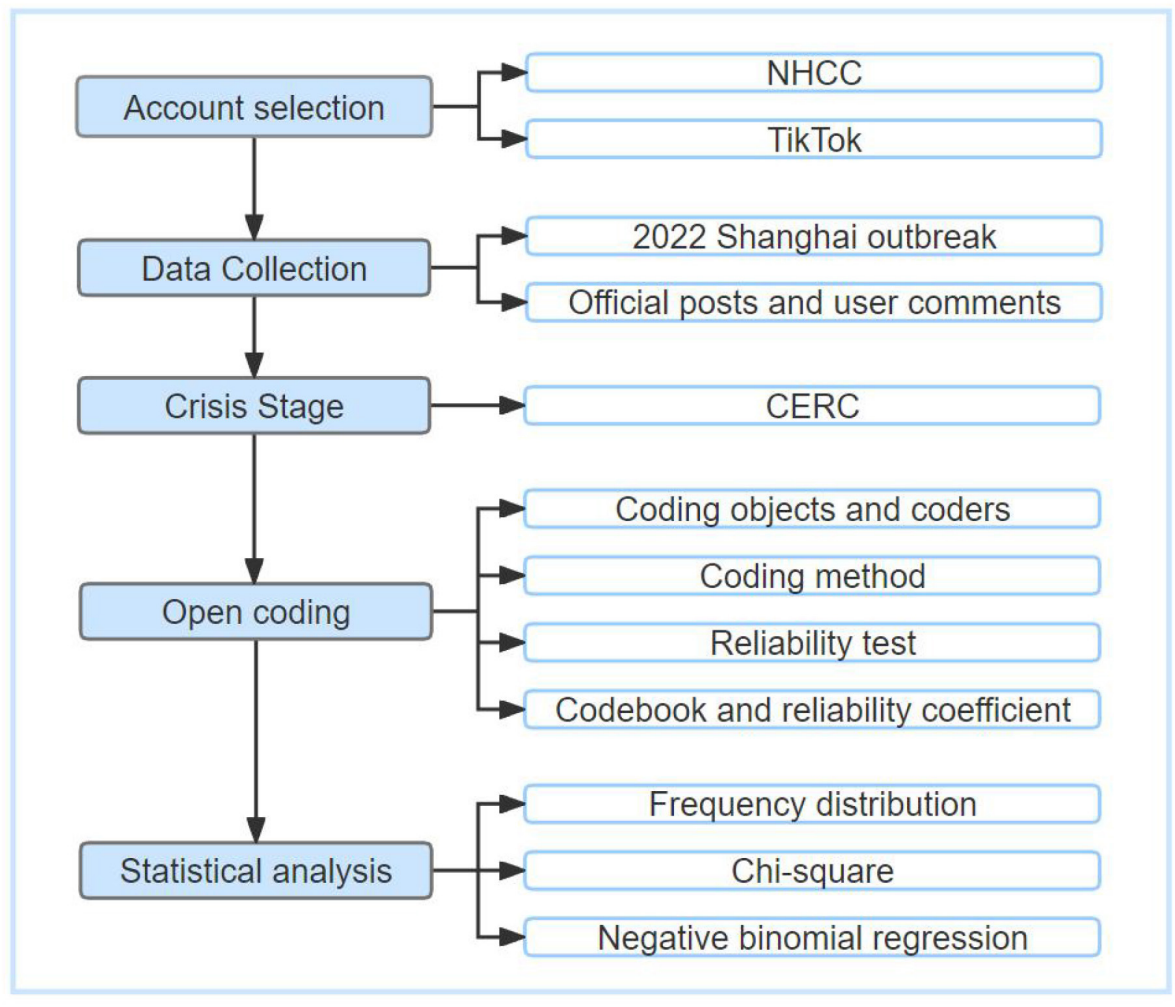
Method flow chart.

The research process of this study is shown in Figure 1. First, we selected the NHCC account on TikTok as the data source and crawled all the posts published by the account during the closure of Shanghai in 2022, as well as the metrics and user comments related to the posts. Second, we used our CERC model-based stage division guide to divide the 2022 Shanghai lockdown into five stages, and the CERC model-based communication strategy guidelines to openly encode the communication strategies for each stage. Third, count classification, descriptive statistics, chi-square validation, and negative binomial regression were employed to evaluate the relationship between stage and communication strategy.

### Account selection

In this study, Healthy China, the NHCC's official account on TikTok, was chosen as the data source. As of August 1, 2022, the account had 4.5 million followers and had posted 2,700 videos, making it the most influential official account on TikTok in the health sector in China.

### Data collection

The data of 527 videos posted by Healthy China from January 1, 2022 to July 14, 2022 were collected. After filtering out data unrelated to COVID-19, 278 videos remained. The video data contained eight types of information: video title, post time, number of views, number of favorites, number of shares, number of comments, and number of likes.

### Crisis stage

**Figure 2:**
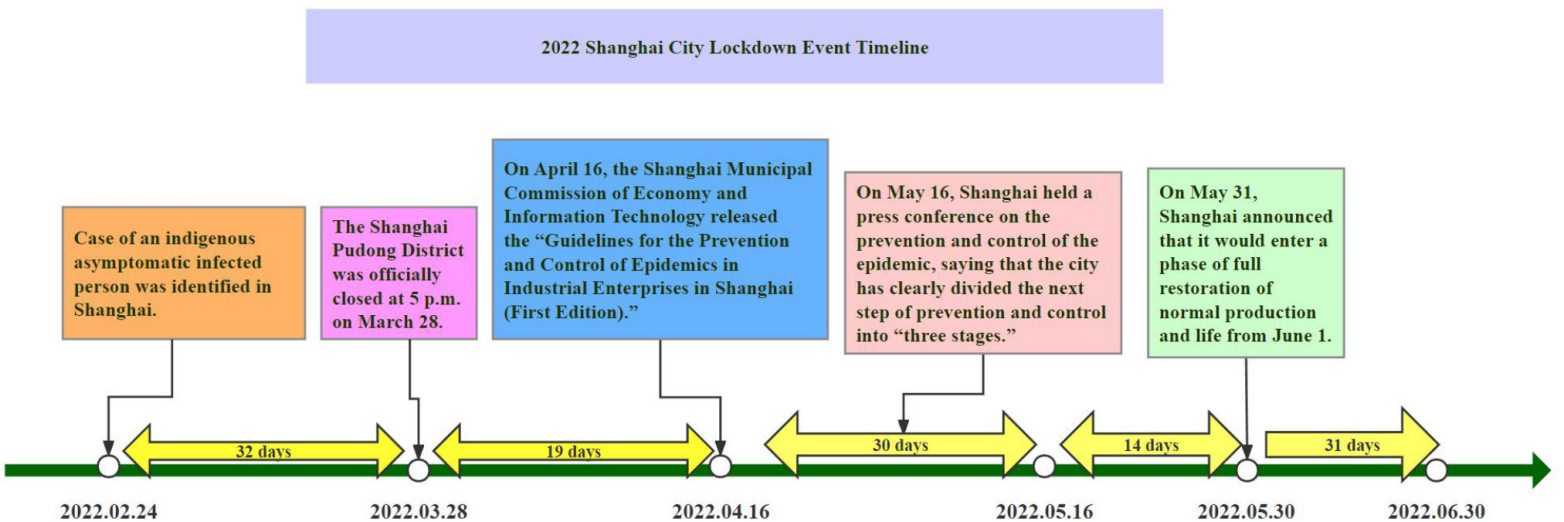
Timeline of the 2022 Shanghai lockdown based on the CERC model.

As shown in Figure 2, the division of stages was based on the entire period of Shanghai's closure in 2022.

The pre-crisis stage was from February 24 to March 27, 2022, when a new case of an indigenous asymptomatic infection was reported in Shanghai.

The initial event stage was from March 28 to April 15, 2022, with the closure of the Shanghai Pudong New Area from 5 pm on March 28, 2022.

The maintenance stage was from April 16 to May 15, 2022. The Shanghai Municipal Commission of Economy and Information Technology issued the “Guidelines for the Prevention and Control of Epidemics in Industrial Enterprises in Shanghai (First Edition)” on April 16, 2022.

The resolution stage lasted from May 16 to 30, 2022. On May 16, 2022, Shanghai held a press conference on the prevention and control of the epidemic, and Shanghai's Vice Mayor Zong Ming stated that with the implementation of various prevention and control measures, Shanghai has clearly divided the next prevention and control work into “three stages.”

The evaluation stage was from May 30 to June 30, 2022. On May 31, 2022, Shanghai announced that it would enter a stage of full restoration of normal production and life from June 1, 2022.

Based on the above assessments, the number of videos was reduced to 207.

### Open coding

#### Coding objects and coders

The communication strategy used for the video was identified by reading the video title and watching its content. As the content of the video was related to the fields of data science and medicine, to ensure high coding validity, two coders were recruited—a PhD student with a data science background and a PhD student with a medical background.

#### Coding method

This study employed an open coding approach. To avoid introducing new classes, a minimal codebook based on the CERC model was created first, and then previous studies' codebooks were integrated. In this case, the current measurements could be more easily compared to previous study results. Finally, repeated coding tests, reliability calculations, and divergence reduction were used to improve coders' perceived consistency of coding standards, and thus coding reliability. Formal coding was not begun until the Holsti's coefficient reached 0.9 or higher.

#### Reliability test

Inter-coder reliability is a measure of study quality. In this study, 30% of the data were randomly selected and coded by two coders who were not allowed to discuss and communicate with each other. If the test results did not meet the minimum reliability criteria, the coding was retested. Prior to retesting, the two coders were trained again, particularly in categories where there were large differences in the coding results.

#### Codebook and reliability coefficient

A combined inter-coder Holsti coefficient of 0.9 was obtained, and the two coders completed the remainder of the coding. Table 1 gives the definitions of the key concepts and Table 2 presents the coded classes and confidence coefficients.

**Table 1:** Definition of key concepts.

**Indicator**	**Definition**
Strategy	Strategy refers to the core strategy of Healthy China communication at this stage, such as reassurance, uncertainty reduction, updates regarding resolution, preparations, self-efficacy, and consensus about lessons and new understandings of risks.
Substrategy	Substrategy refers to the information type composition implementing the core strategy. For example, uncertainty reduction includes new cases per day, nucleic acid testing, vaccines, epidemic situation, and policy.
Operational definitions	Operational definitions are the operability criteria used in this study to classify posts.
Examples	Examples show the instances that conform to a substrategy.
Intercoder reliability	Intercoder reliability is a quantitative indicator that indicates the degree to which multiple independent coders in a coding job make consistent judgments about the characteristics of the same piece of information or record content.

**Table 2:** Coded strategy categories, operation definitions, examples, and confidence coefficients.

**Strategies (substrategies)**	**Operational definitions**	**Examples**	**Intercoder reliability**
**Reassurance**			1.00
Government Statement	Government spokespersons convey the government's thinking to the public through press conferences	Adhering to the general policy of “dynamic zero” without wavering.	1.00
**Uncertainty reduction**			1.00
New cases per day	Number of new confirmed cases per day	At 24:00 on February 27, there were 234 new confirmed cases, including 87 indigenous cases.	1.00
Nucleic acid testing	Answering concerns about nucleic acid testing	What is the difference between antigen testing and nucleic acid testing?	1.00
Vaccines	Answering concerns about vaccines	What should people be aware of, in terms of vaccination and personal protection, in the face of the Omicron variant?	1.00
Epidemic situation	Answering concerns about the status of the epidemic	How has the national epidemiological situation changed in the last few days?	1.00
Policy	Answering concerns about the anti-epidemic policy	What is the reason for the centralized isolation and management of mild cases?	1.00
**Updates regarding resolution**			1.00
Prevention and control programs	Answering concerns about the new vaccination program	The third edition of the nucleic acid testing guidelines calls for the delineation of precise testing. Does this mean that mass nucleic acid testing of the entire workforce will not be done in the future?	1.00
**Preparations**			1.00
Transportation	Travel-related preparations	How can the transport sector do a good job of providing transport services for students and workers on their return journey and of preventing and controlling the epidemic?	1.00
Medical needs of the general population	Medical information relevant to the general population	How do you ensure access to health care for people in areas where outbreaks occur?	1.00
Living goods	Problems with the supply of subsistence goods	What specific measures has the Ministry of Commerce instructed the regions concerned to take to ensure the supply of living materials in the key areas of epidemic prevention and control?	1.00
Input risk	Information relating to the control of imported cases from overseas	How can customs further reduce the risk of overseas importation of outbreaks?	1.00
Students	Information relating to students	In the current epidemic situation, how can people accurately prevent and control the epidemic and ensure that campus recruitment activities are held in a safe and orderly manner?	1.00
Vaccine development	Information related to vaccination development status	What is the progress of the new coronavirus vaccination? In particular, what is the status of the booster vaccination?	1.00
**Self-efficacy**			1.00
Personal protection	Information relating to personal protection	Do this to travel safely and in a healthy manner during the epidemic.	1.00
**Consensus about lessons and new understandings of risks**			1.00
Negative case	Misconduct in the fight against the epidemic by local authorities	Notification of typical cases of violation of the “Nine No's.”	1.00

### Statistical analysis

Counts and percentages were used to summarize categorical variables to understand the distribution of strategies across stages. Descriptive statistical analysis was performed to investigate the distribution of public engagement indicators, and the relationship between stage and communication strategy was assessed by performing chi-squared tests and negative binomial regression analysis.

## Results

### Frequency distribution of strategies

Table 3 shows that in terms of stages, Healthy China posted the most posts in the evaluation (28.50%) and pre-crisis (26.57%) stages, followed by maintenance (20.29%), initial event (14.98%), and resolution (9.66%) stages.

**Table 3:** Distribution of communication strategies in different stages.

**Stage**	**Frequency**	**Percent (%)**
Evaluation	59	28.50
Pre-crisis	55	26.57
Maintenance	42	20.29
Initial event	31	14.98
Resolution	20	9.66
Total	207	100.0

Table 4 shows that uncertainty reduction (65.70%) was the most used strategy, followed by preparations (13.04%), reassurance (10.63%), updates regarding resolution (4.83%), self-efficacy (3.86%), and consensus about lessons (1.93%).

**Table 4:** Percentage of communication strategies.

**Strategy**	**Frequency**	**Percent (%)**
Uncertainty reduction	136	65.70
Preparations	27	13.04
Reassurance	22	10.63
Updates regarding resolution	10	4.83
Self-efficacy	8	3.86
Consensus about lessons	4	1.93
Total	207	100.0

As shown in Table 5, in terms of substrategies, new cases per day were ranked first at 38.16%. Other substrategies comprising more than 5% of the total were epidemic situations (11.59%), government statements (10.63%), policies (9.66%), and students (6.28%).

**Table 5:** Frequency distribution of communication substrategies.

**Substrategy**	**Frequency**	**Percent (%)**
New cases per day	79	38.16
Epidemic situation	24	11.59
Government statement	22	10.63
Policy	20	9.66
Student	13	6.28
Prevention and control program	10	4.83
Personal protection	8	3.86
Vaccine	7	3.38
Nucleic acid testing	6	2.90
Negative case	4	1.93
Transportation	4	1.93
Vaccine development	3	1.45
Input risk	3	1.45
Medical needs of the general population	2	0.97
Living goods	2	0.97
Total	207	100.0

Overall, Healthy China posted the most videos in the evaluation (28.50%) and pre-crisis (26.57%) stages, with 65.70% of the videos aimed at uncertainty reduction. New cases per day were the most widely used substrategies, accounting for 38.16% of all substrategies.

### Descriptive analysis of public engagement

The descriptive analysis uses means or medians to describe the overall data. Table 6 shows that the maximum values of number of views, number of favorites, number of shares, number of comments, and number of likes exceeded the mean by three standard deviations, indicating that the data fluctuated considerably, and that it was more appropriate to use the median than the mean to describe the overall level.

**Table 6:** Descriptive distribution of public engagement metrics.

**Engagement Metrics**	**Count**	**Min**.	**Max**.	**Mean**	**Standard deviation**	**Median**
Number of views	152	9,649.000	1,182,605.000	105,714.289	134,221.961	69,627.500
Number of favorites	207	0.000	958.000	56.493	103.827	30.000
Number of shares	207	0.000	3,725.000	100.348	307.975	28.000
Number of comments	207	0.000	1,765.000	38.575	145.141	12.000
Number of likes	207	20.000	19,223.000	1,160.420	2,140.592	460.000

Table 7 shows the medians of the five public engagement indicators for videos released by Healthy China at different stages. Pre-crisis was found to rank first in all indicators, with 120,758,500 views, 760,000 likes, 51,000 favorites, 87,000 shares, and 20,000 comments.

**Table 7:** Median distribution of public engagement indicators at different stages.

**Engagement metrics**	**Stage**					**Total**
	**Pre-crisis**	**Initial event**	**Maintenance**	**Evaluation**	**Resolution**	
Number of views	120,758.500	74,654.000	56,702.500	45,440.5	76,200.000	69,627.500
Number of likes	760.000	359.000	214.500	412	710.000	460.000
Number of favorites	51.000	39.000	20.500	18	30.500	30.000
Number of shares	87.000	45.000	20.500	14	21.000	28.000
Number of comments	20.000	12.000	7.000	12	10.500	12.000

### Substrategies with a high level of public engagement at each stage

As shown in Table 8, in terms of stage, in pre-crisis, government statements had the highest number of views; new cases per day had the highest number of likes and shares; and vaccines had the highest number of comments and likes. In initial event, vaccine development had the highest value of all the indicators. In maintenance, videos with medical need topics of the general population had the highest number of views, likes, shares, and comments, whereas government statements had the highest number of likes. In resolution, new cases per day had the highest number of views and likes; policies had the highest number of shares; and government statements had the highest number of comments and likes. In evaluation, personal protection ranked first in terms of number of views, favorites, shares, and likes, followed by prevention and control programs, with the highest number of comments.

**Table 8:** Median distribution of public engagement indicators for communication substrategies at different stages.

**Substrategy**	**Stage**	**Number of views**	**Number of favorites**	**Number of shares**	**Number of comments**	**Number of likes**
Personal protection	Pre-crisis	16,048.000	23.000	37.000	26.000	672.000
	Initial event	0.000	11.000	45.000	5.000	55.000
	Maintenance	38,915.000	20.000	36.000	25.000	868.000
	Resolution	0.000	0.000	0.000	0.000	0.000
	Evaluation	110,303.000	87.000	100.000	40.000	1,157.000
	Total	38,915.000	21.500	41.000	25.500	770.000
Transportation	Pre-crisis	0.000	3.000	8.000	0.000	54.000
	Initial event	0.000	0.000	0.000	0.000	0.000
	Maintenance	24,814.000	29.000	30.000	17.000	673.000
	Resolution	0.000	0.000	0.000	0.000	0.000
	Evaluation	0.000	0.000	0.000	0.000	0.000
	Total	24,814.000	27.500	26.500	10.000	452.500
Negative case	Pre-crisis	0.000	0.000	0.000	0.000	0.000
	Initial event	0.000	0.000	0.000	0.000	0.000
	Maintenance	0.000	0.000	0.000	0.000	0.000
	Resolution	0.000	0.000	0.000	0.000	0.000
	Evaluation	20,967.000	18.500	47.000	37.000	611.000
	Total	20,967.000	18.500	47.000	37.000	611.000
Student	Pre-crisis	64,387.000	28.500	61.500	37.500	233.000
	Initial event	0.000	0.000	0.000	0.000	0.000
	Maintenance	23,060.000	13.500	19.000	6.500	666.000
	Resolution	0.000	0.000	0.000	0.000	0.000
	Evaluation	19,432.500	8.000	11.000	5.000	253.000
	Total	21,493.500	8.000	12.000	5.000	253.000
Government statement	Pre-crisis	368,245.000	60.500	66.000	12.500	638.000
	Initial event	83,539.500	29.000	58.000	9.000	488.000
	Maintenance	69,731.000	89.000	78.000	36.500	3,856.000
	Resolution	28,412.500	31.500	34.000	35.500	2,234.000
	Evaluation	25,270.000	12.000	14.000	2.000	363.000
	Total	72,192.500	28.500	36.000	10.000	494.500
Policy	Pre-crisis	87,776.000	52.500	82.500	34.500	1,695.500
	Initial event	66,750.500	56.000	73.000	44.000	802.000
	Maintenance	69,972.500	45.000	108.000	29.000	431.000
	Resolution	76,200.000	26.000	35.500	4.000	194.000
	Evaluation	23,843.500	2.000	12.000	1.000	37.000
	Total	66,557.000	29.500	50.500	15.000	357.500
Medical needs of the general population	Pre-crisis	49,883.000	45.000	61.000	32.000	430.000
	Initial event	0.000	0.000	0.000	0.000	0.000
	Maintenance	131,272.000	156.000	167.000	59.000	2,293.000
	Resolution	0.000	0.000	0.000	0.000	0.000
	Evaluation	0.000	0.000	0.000	0.000	0.000
	Total	90,577.500	100.500	114.000	45.500	1,361.500
Nucleic acid testing	Pre-crisis	0.000	6.000	27.000	0.000	84.000
	Initial event	0.000	0.000	0.000	0.000	0.000
	Maintenance	0.000	9.000	16.000	5.000	47.000
	Resolution	0.000	0.000	0.000	0.000	0.000
	Evaluation	0.000	5.000	14.000	0.500	43.500
	Total	0.000	6.000	16.000	0.500	45.500
New cases per day	Pre-crisis	175,978.000	64.500	162.000	17.000	1,072.000
	Initial event	115,809.500	27.500	13.500	7.500	216.500
	Maintenance	63,140.500	19.500	12.500	6.000	178.500
	Resolution	136,036.000	75.000	31.000	32.000	1,713.000
	Evaluation	79,064.000	38.000	14.500	15.500	1,039.000
	Total	122,983.000	46.000	24.000	12.000	760.000
Living goods	Pre-crisis	0.000	0.000	0.000	0.000	0.000
	Initial event	58,664.000	51.500	53.000	34.500	668.000
	Maintenance	0.000	0.000	0.000	0.000	0.000
	Resolution	0.000	0.000	0.000	0.000	0.000
	Evaluation	0.000	0.000	0.000	0.000	0.000
	Total	58,664.000	51.500	53.000	34.500	668.000
Epidemic situation	Pre-crisis	61,119.500	42.500	54.000	9.500	578.500
	Initial event	40,052.500	39.000	63.500	23.500	733.000
	Maintenance	38,812.000	7.000	28.000	4.500	72.000
	Resolution	0.000	5.000	14.000	4.000	57.000
	Evaluation	20,087.000	3.000	10.000	4.000	34.000
	Total	43,402.000	15.500	36.500	8.000	376.500
Vaccine	Pre-crisis	146,001.500	58.000	97.000	43.000	2,865.000
	Initial event	49,422.500	54.000	112.500	34.000	1,223.500
	Maintenance	0.000	1.000	18.000	2.000	47.000
	Resolution	15,038.000	13.000	18.000	14.000	110.000
	Evaluation	0.000	0.000	0.000	0.000	0.000
	Total	45,469.000	39.000	40.000	19.000	926.000
Vaccine development	Pre-crisis	28,443.000	29.000	37.000	19.500	1,906.000
	Initial event	176,657.000	231.000	401.000	64.000	1,947.000
	Maintenance	0.000	0.000	0.000	0.000	0.000
	Resolution	0.000	0.000	0.000	0.000	0.000
	Evaluation	0.000	0.000	0.000	0.000	0.000
	Total	28,589.000	39.000	47.000	23.000	1,947.000
Input risk	Pre-crisis	0.000	0.000	0.000	0.000	0.000
	Initial event	0.000	0.000	0.000	0.000	0.000
	Maintenance	0.000	0.000	0.000	0.000	0.000
	Resolution	15,119.500	11.000	16.000	1.000	119.000
	Evaluation	0.000	0.000	0.000	0.000	0.000
	Total	15,119.500	11.000	16.000	1.000	119.000
Prevention and control program	Pre-crisis	45,808.500	39.000	78.000	35.500	1,553.000
	Initial event	0.000	0.000	0.000	0.000	0.000
	Maintenance	0.000	0.000	0.000	0.000	0.000
	Resolution	0.000	0.000	0.000	0.000	0.000
	Evaluation	33,470.500	21.500	29.500	53.500	546.500
	Total	37,435.500	33.500	50.000	50.000	687.000
Total		69,627.500	30.000	28.000	12.000	460.000

### Chi-square test

The chi-square test was performed to investigate the differential relationship between the stages and substrategies. As shown in Table 9, stage presented a 0.01 level of significance for substrategies (chi = 109.904, *p* = 0.000 < 0.01). By comparing the differences in percentages, percentage of government statements in the initial event was found to be 16.13%, significantly higher than the average of 10.63%. The percentage of new cases per day in the resolution stage was 45.00%, also significantly higher than the average of 38.16%. The percentage of epidemic situations in pre-crisis was 18.18%, significantly higher than the average of 11.59%.

**Table 9:** Chi-square analysis of stage to substrategy.

**Substrategy**	**Stage (%)**					**Total**	**χ2**	***p***
	**Evaluation**	**Initial event**	**Maintenance**	**Pre-crisis**	**Resolution**			
Personal protection	1 (1.69)	1 (3.23)	5 (11.90)	1 (1.82)	0 (0.00)	8 (3.86)		
Transportation	0 (0.00)	0 (0.00)	3 (7.14)	1 (1.82)	0 (0.00)	4 (1.93)		
Negative case	4 (6.78)	0 (0.00)	0 (0.00)	0 (0.00)	0 (0.00)	4 (1.93)		
Student	9 (15.25)	0 (0.00)	2 (4.76)	2 (3.64)	0 (0.00)	13 (6.28)		
Government statement	5 (8.47)	5 (16.13)	4 (9.52)	6 (10.91)	2 (10.00)	22 (10.63)		
Policy	7 (11.86)	4 (12.90)	3 (7.14)	4 (7.27)	2 (10.00)	20 (9.66)		
Medical needs of the general population	0 (0.00)	0 (0.00)	1 (2.38)	1 (1.82)	0 (0.00)	2 (0.97)	109.904	0.000^**^
Nucleic acid testing	4 (6.78)	0 (0.00)	1 (2.38)	1 (1.82)	0 (0.00)	6 (2.90)		
New cases per day	20 (33.90)	12 (38.71)	18 (42.86)	20 (36.36)	9 (45.00)	79 (38.16)		
Living goods	0 (0.00)	2 (6.45)	0 (0.00)	0 (0.00)	0 (0.00)	2 (0.97)		
Epidemic situation	3 (5.08)	4 (12.90)	4 (9.52)	10 (18.18)	3 (15.00)	24 (11.59)		
Vaccine	0 (0.00)	2 (6.45)	1 (2.38)	3 (5.45)	1 (5.00)	7 (3.38)		
Vaccine development	0 (0.00)	1 (3.23)	0 (0.00)	2 (3.64)	0 (0.00)	3 (1.45)		
Input risk	0 (0.00)	0 (0.00)	0 (0.00)	0 (0.00)	3 (15.00)	3 (1.45)		
Prevention and control program	6 (10.17)	0 (0.00)	0 (0.00)	4 (7.27)	0 (0.00)	10 (4.83)		
Total	59	31	42	55	20	207		

The symbol

** indicates the value of *p* < 0.01.

In conclusion, all substrategies showed significant differences across stage.

### Negative binomial regression

Table 10 shows the negative binomial regression results for the effects of substrategies and stage on public engagement indicators.

**Table 10:** Negative binomial regression of substrategy and stage on public engagement indicators.

**Item**	**Regression coefficient**				
**Variables**	**Number of views**	**Number of favorites**	**Number of shares**	**Number of comments**	**Number of likes**
Intercept	11.224^**^ (43.291)	3.269^**^ (14.847)	3.828^**^ (17.457)	32.818^**^ (149.291)	6.428^**^ (29.511)
Substrategy	−0.039 (−1.580) *p =* 0.114 OR = 0.962	−0.002 (−0.077) *p =* 0.938 OR = 0.998	−0.077^**^ (−3.544) *p =* 0.000 OR = 0.926	−1.711^**^ (−78.489) *p =* 0.000 OR = 0.181	−0.039 (−1.816) *p =* 0.069 OR = 0.962
Stage	0.223^**^ (3.761) *p =* 0.000 OR = 1.250	0.263^**^ (5.068) *p =* 0.000 OR = 1.301	0.448^**^ (8.644) *p =* 0.000 OR = 1.566	−1.445^**^ (−27.993) *p =* 0.000 OR = 0.236	0.313^**^ (6.087) *p =* 0.000 OR = 1.368
Sample size	152	207	207	207	207
Likelihood ratio	χ2 (2) = 15.103, *p =* 0.001	χ2 (2) = 23.430, *p =* 0.000	χ2 (2) = 72.729, *p =* 0.000	χ2 (2) = −4,342.565, *p =* 1.000	χ2 (2) = 36.498, *p =* 0.000
McFadden *R*^2^	0.004	0.011	0.031	−2.248	0.011

** p < 0.01, z-values in brackets.

The order of stages and substrategies mentioned in the results below is shown as follows.

Stage: Evaluation, initial event, maintenance, pre-crisis, and resolution.

Substrategy: Personal protection, transportation, negative case, student, government statement, policy, medical needs of the general population, nucleic acid testing, new cases per day, living goods, epidemic situation, vaccine, vaccine development, input risk, and prevention and control program.

#### Number of views

As per the results, the regression coefficient of the substrategy was −0.039, which was not statistically significant (z = −1.580, *p* = 0.114 > 0.05), implying that the substrategy did not have an impact on the number of plays.

The regression coefficient for the stage was 0.223, statistically significant at the 0.01 level (z = 3.761, *p* = 0.000 < 0.01), implying that the stage had a statistically significant positive effect on number of views. An odds ratio (OR) of 1.250 means that for each unit increase in stage, the change (increase) in number of views was 1.250 times greater.

The summary analysis showed that stage had a significant positive impact on number of views. However, the substrategy did not affect number of views.

#### Number of favorites

The regression coefficient value of the substrategy was −0.002, but it was not significant (z = −0.077, *p* = 0.938 > 0.05), which means that the substrategy did not have an impact on the number of favorites.

The regression coefficient value of the stage was 0.263, which was significant at the 0.01 level (z = 5.068, *p* = 0.000 < 0.01), which means that the stage had a significant positive impact on number of favorites. Moreover, the OR was 1.301, which means that when the stage increased by one unit, the change (increase) of the number of favorites was 1.301 times.

The summary analysis showed that the stage had a significant positive impact on number of favorites. However, the substrategy did not affect the number of favorites.

#### Number of shares

The regression coefficient value of substrategy was −0.077, which was significant at the 0.01 level (z = −3.544, *p* = 0.000 < 0.01), meaning that substrategy had a significant negative impact on the number of shares. Moreover, the OR value was 0.926, which means that when the substrategy increased by one unit, the change (decrease) in the number of shares was 0.926 times.

The regression coefficient value of the stage was 0.448, which was significant at the 0.01 level (z = 8.644, *p* = 0.000 < 0.01). This means that the stage had a significant positive impact on the number of shares. Furthermore, OR was 1.566, which means that when the stage increased by one unit, the change (increase) of the number of shares was 1.566 times.

The summary analysis showed that the stage had a significant positive impact on the number of shares, and substrategy had a significant negative impact on the number of shares.

#### Number of comments

The regression coefficient value of the substrategy was −1.711, which was significant at the 0.01 level (z = −78.489, *p* = 0.000 < 0.01). Hence, the substrategy had a significant negative impact on the number of comments. Moreover, the OR value was 0.181, which means that when the substrategy increased by one unit, the change (decrease) of the number of comments was 0.181 times.

The regression coefficient value of the stage was −1.445, which was significant at the 0.01 level (z = −27.993, *p* = 0.000 < 0.01). Hence, the stage had a significant negative impact on the number of comments. Furthermore, the OR was 0.236, which means that when the stage increased by one unit, the change (decrease) of the number of comments was 0.236 times.

The summary analysis showed that both the substrategy and stage significantly impacted the number of comments.

#### Number of likes

The regression coefficient value of the substrategy was −0.039, but it was not significant (z = −1.816, *p* = 0.069 > 0.05). Hence, the substrategy did not affect the number of likes.

The regression coefficient value of the stage was 0.313, which was significant at the 0.01 level (z = 6.087, *p* = 0.000 < 0.01). Hence, the stage had a significant positive impact on the number of likes. Moreover, the OR was 1.368, which means that when the stage increased by one unit, the change (increase) of the number of likes was 1.368 times.

The summary analysis showed that the stage had a significant positive impact on the number of likes, but substrategy did not impact the number of likes.

## Discussion

### Findings

This study is the first to explore how the PHA communicates with the public on TikTok in the context of the normalization of COVID-19.

First, based on the CERC model and the results of the chi-square test, it was found that Healthy China did not follow the CERC model's proposal to use the corresponding communication strategies in the specified stages (e.g., use of communication strategies belonging to the pre-crisis stage in the pre-crisis stage itself), but instead used a mixture of communication strategies belonging to other stages (the communication strategy originally belonging to the initial event stage was used in the pre-crisis stage). Specifically, Healthy China used uncertainty reduction, reassurance, and self-efficacy strategies in the pre-crisis stage, which was originally part of the initial event stage, and updates regarding resolution, which was originally part of the resolution stage. The current finding is in line with the results of Alhassan et al., indicating that information related to uncertainty reduction, reassurance, and effectiveness is prevalent in all stages (21).

The developers of the CERC model argued that some potential crises and emergencies might not follow the sequence of pre-crisis, initial event, maintenance, resolution, and evaluation due to a variety of factors (20). The current findings extend this idea; some potential crises and emergencies may not follow the conventional sequence in the context of epidemic normalization, during which, communication strategies are more flexible and do not adhere to the CERC model.

In the context of the normalization of COVID-19, the government and medical institutions must update their existing medical programs periodically to respond to new variants of COVID-19 and to constantly reflect on past shortcomings in the fight against the epidemic. Furthermore, the public must update their knowledge of the virus and prepare their responses in advance to prevent their work, studies, and lives from being disrupted by a new outbreak of COVID-19. This may lead to a new outbreak cycle in which the PHA may issue communication strategies such as uncertainty reduction and updates regarding resolution during the pre-crisis stage.

Second, compared to the communication strategy in the early period of COVID-19, the strategy in 2022 is significantly different. Specifically, uncertainty reduction (65.70%) was found to be the most commonly used strategy in this study, with new cases per day being the predominant substrategy, accounting for 38.16% of all substrategies. Li et al. studied eight public health and UN agencies, and the data showed that videos with a focus on individual prevention were the most prevalent (16). Furthermore, Healthy China posted the most videos during the evaluation (28.50%) and pre-crisis (26.57%) stages, whereas the official Twitter account of the Saudi Arabian Ministry of Health posted the majority of tweets during the initial event stage (65.89%) (21). Considering that the data from the previous study came from the early days of COVID-19, it is evident that the focus of PHA's communication has changed in the context of the normalization of COVID-19. It is likely that in 2020 and 2021, Chinese scientists, medical scientists, and the public were all ignorant of COVID-19; therefore, how to be safe from infection was the most critical health information. However, with the normalization of COVID-19, the vaccination rate has reached a high level, and people are not worried that they will be infected with COVID-19. On the contrary, people are more concerned about whether the recurrence of the epidemic will hinder their lives and travel. Therefore, policies to reduce uncertainties must be prioritized.

Third, the median indicator for the pre-crisis stage was the highest in terms of number of views, number of likes, number of favorites, number of shares, and number of comments, indicating a high level of public engagement during the pre-crisis stage (21). This may be because the government statements in the pre-crisis period seemed more attractive to the public. As government statements have a high degree of stability, direction, and guidance, the public tended to draw from government videos during that stage if they encountered problems related to COVID-19 in their work and life. Chen et al. found that on TikTok, the public was more interested in government posts about crisis management, which is supported by the current results (5).

### Limitations

In this study, we discovered that Healthy China did not use communication strategies in accordance with the CERC model's strategic guidelines. However, we were unable to determine whether this behavior should be encouraged; therefore, future research should focus on sentiment analysis and topic models to further analyze these communication strategies.

### Conclusions

This study investigated how Healthy China communicates with the public in the context of the normalization of COVID-19 based on the CERC theory, using the 2022 Shanghai city closure due to the COVID-19 outbreak as a timeline and NHCC's official account of Healthy China on TikTok as a data source. The findings were as follows:

First, based on the results of the CERC model and the chi-square test, it was discovered that Healthy China was more flexible in choosing communication strategies than the CERC model suggested.

Second, the communication strategies used by Healthy China during the normalization of COVID-19 were markedly different. Healthy China's main aim in releasing the video was to reduce uncertainty, with new cases per day being the most used substrategy.

Third, there was a clear variation in the strategies used by Healthy China at different stages.

Fourth, the public was more likely to engage with Healthy China video during the pre-crisis stage, reflecting the changing public demand for information about the outbreak.

Finally, the stages had significant positive effects on number of views, number of favorites, number of likes, and number of shares. The current findings provide insights for governments and PHA to communicate with the public in the context of COVID-19 normalization. Furthermore, these findings help the government or PHA in reducing the number of controversial topics that arise and increasing the amount of video content that makes the public feel as at ease as possible.

## Data availability statement

The raw data supporting the conclusions of this article will be made available by the authors, without undue reservation.

## Author contributions

SC: conceptualization, methodology, and software. SZ: writing-original draft preparation. JK: writing-reviewing, supervision, and editing. All authors contributed to the article and approved the submitted version.

## Funding

This work was supported by a National Research Foundation of Korea (NRF) grant funded by the Korean Government (NRF-2020R1A2C1014957).

## Conflict of interest

The authors declare that the research was conducted in the absence of any commercial or financial relationships that could be construed as a potential conflict of interest.

## Publisher's note

All claims expressed in this article are solely those of the authors and do not necessarily represent those of their affiliated organizations, or those of the publisher, the editors and the reviewers. Any product that may be evaluated in this article, or claim that may be made by its manufacturer, is not guaranteed or endorsed by the publisher.
